# Ethyl acetate fraction from *Angelica sinensis* inhibits IL-1β-induced rheumatoid synovial fibroblast proliferation and COX-2, PGE2, and MMPs production

**DOI:** 10.1186/0717-6287-47-41

**Published:** 2014-09-05

**Authors:** Won-Seok Lee, Jin-Han Lim, Myung-Soon Sung, Eun-Gyeong Lee, Yoo-Jeong Oh, Wan-Hee Yoo

**Affiliations:** Department of Internal Medicine, Division of Rheumatology, Chonbuk National University Medical School and Research Institute of Clinical Medicine of Chonbuk National University Hospital-Chonbuk National University, San 2-20 Geumam-dong, Deokjin-gu, Jeonju, Jeonbuk, 561-180 South Korea

**Keywords:** *Angelica sinensis*, COX, IL-1β, MMPs, Rheumatoid arthritis (RA)

## Abstract

**Background:**

The root of *Angelica sinensis* (AS), also known as “Dang-gui,” was a popular herbal medicine widely used in the treatment of gynecological diseases in China, Korea, and Japan for a long time. This study aimed to determine the effects of ethyl acetate fraction from *Angelica sinensis* (EAAS) on the interleukin-1β (IL-1β)-induced proliferation of rheumatoid arthritis synovial fibroblasts (RASFs), and production of matrix metalloproteinases (MMPs), cyclooxygenase (COX) 2, and prostaglandin E2 (PGE2), involved in articular bone and cartilage destruction, by RASFs.

**Results:**

RASF proliferation was evaluated with cholecystokinin octapeptide (CCK-8) reagent in the presence of IL-1β with/without EAAS. Expression of MMPs, tissue inhibitor of metalloproteinases-1 (TIMP-1), COXs, PGE2, and intracellular mitogen-activated protein kinase (MAPK) signaling molecules, including p-ERK, p-p38, p-JNK, and NF-κB, were examined using immunoblotting or semi-quantitative reverse transcription-polymerase chain reaction and enzyme-linked immunosorbent assay. EAAS inhibited IL-1β-induced RASF proliferation; MMP-1, MMP-3, and COX-2 mRNA and protein expressions; and PGE2 production. EAAS also inhibits the phosphorylation of ERK-1/2, p38, and JNK, and activation of NF-κB by IL-1β.

**Conclusion:**

EAAS might be a new therapeutic modality for rheumatoid arthritis management.

## Background

Rheumatoid arthritis (RA) is a chronic inflammatory disease characterized by robust infiltration of leukocytes into the synovium, resulting in hyperplasia of the synovial lining, progressive cartilage destruction, and finally, erosion of the underlying bone [1]. Synovial fibroblasts mediate joint destruction in RA by producing inflammatory mediators such as cytokines, matrix metalloproteinases (MMPs), and cyclooxygenase (COX)-2 that facilitate the expansion and invasion of synovial fibroblasts into the adjacent tissue. The regulation of these events has been a primary target of therapeutic intervention in RA [2]. Interleukin 1β (IL-1β) is considered the most important cytokine in the pathogenic process of inflammation in RA; it induces proliferation of rheumatoid arthritis synovial fibroblasts (RASFs), and production of high levels of MMPs and prostaglandin E2 (PGE2) via COX expression by RASFs [3].

The root of *Angelica sinensis* (Oliv.) Diels (Apiaceae) (AS), also known as “Dang-gui,” was a popular herbal medicine widely used in the treatment of gynecological diseases in China, Korea, and Japan for a long time. Chemical and pharmacological studies of various extracts or compounds obtained from this herb were found to increase myocardial blood flow, reduce radiation damages [4–6]. It was also demonstrated as mainly consisting of polysaccharides and having a protective effect on gastrointestinal damage and hepatic injury [7, 8]. Recently, the ethyl acetate extract of AS (EAAS) showed higher inhibitory activity of NF-κB transactivation than hexane or water fractions [9]. However, no study has investigated the effects of EAAS on inflammatory reactions, including proliferation of RASFs and production of PGE2 by RASFs, which play a crucial role in the pathogenesis of synovitis in RA.

The present study was undertaken to study the effects of EAAS on IL-1β-induced production of proinflammatory mediators by RASFs. MMPs, PGE2 and COX-2 were studied and intracellular signaling factors were evaluated to identify the mechanisms of the effects of EAAS. Here we showed that EAAS can inhibit IL-1β-induced proliferation and inflammatory reactions via mitogen activated protein kinase (MAPK)/nuclear factor-κB (NF-κB) pathways in RASFs.

## Results

### EAAS inhibits IL-1β-induced proliferation of RASFs

**Figure 1:**
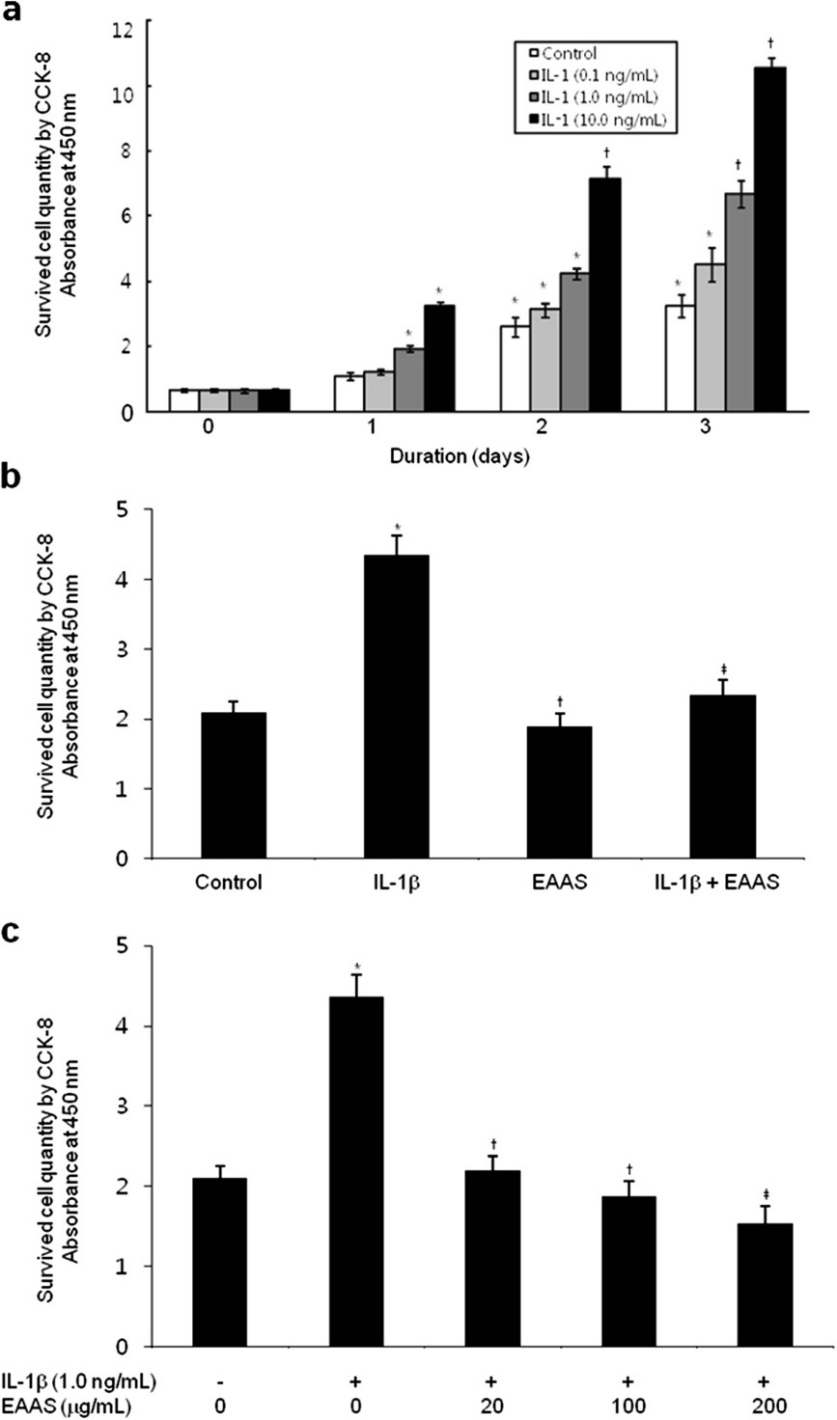
**IL-1β increased the proliferation of RASFs dose-dependently and EAAS inhibits IL-1β-induced proliferation of RASFs.** IL-1β accelerated the proliferation of RASFs dose- and time-dependently (from 0.1 to 10 ng/mL). Results are presented as mean ± SD, n = 3. *p < 0.05 or †p < 0.01 versus day 0 **(a)**. EAAS (100 μg/mL) significantly inhibited IL-1-induced proliferation of RASFs after 2, 3 days culture (p < 0.05) *p < 0.05 versus no IL-1β and EAAS, †p < 0.05 versus no IL-1β and EAAS, ‡p < 0.05 versus IL-1β without EAAS **(b)**. EAAS inhibited IL-1β-induced proliferation of RASFs dose-dependently. *p < 0.05 versus no IL-1β, †p < 0.05, ‡p < 0.01 versus IL-1β without EAAS **(c)**.

To evaluate the effect of EAAS on the growth properties of RASFs, we initially measured cell proliferation with IL-1β for 3 days. IL-1β is well known as a potent growth-promoting factor for RASFs. Cell proliferation was assayed as described in Materials and Methods. As shown in Figure 1a, IL-1β accelerated the proliferation of RASFs dose-dependently (from 0.1 to 10 ng/mL) over time. To know the effect of EAAS on IL-1β-induced proliferation of RASFs, EAAS (100 μg/mL) was added to the RASFs cultures with/without IL-1β (1.0 ng/mL) for 2 days, and cholecystokinin octapeptide (CCK-8) assay was performed. As shown in Figure 1b, the increase in RASF proliferation was significantly greater in the culture with IL-1β than that in the control without IL-1β and EAAS (*p <* 0.05). In Figure 1c, to ascertain the dose-dependent effect of EAAS on IL-1β-induced proliferation of RASFs, different doses of EAAS (20, 100, 200 μg/mL) were added to the RASFs cultures with IL-1β (1.0 ng/mL) for 2 days, and CCK-8 assay was performed.

### Effect of EAAS on IL-1β-induced MMPs, TIMPs, and COX mRNA expressions in RASFs

**Figure 2:**
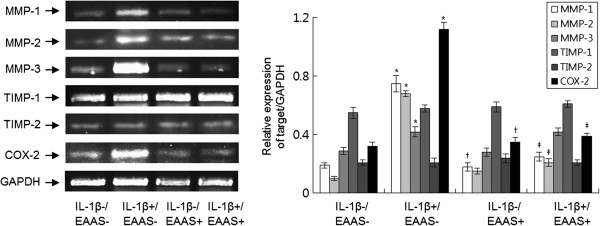
**EAAS inhibited IL-1β-induced production of MMPs and COX-2 in RASFs.** IL-1β enhanced the expression of MMP-1, MMP −3, TIMP-1, and COX-2 protein compared with the results without IL-1β. EAAS inhibited the IL-1β-induced expression of MMP-1, MMP-3, and COX-2 protein. Results are presented as mean ± SD. n = 3. *p < 0.05 versus no IL-1β and EAAS, †p < 0.05 versus no IL-1β and EAAS, ‡p < 0.05 versus IL-1β without EAAS.

Real-time PCR was performed to evaluate the expressions of MMP-1, MMP-3, Tissue inhibitor of metalloproteinase (TIMP)-1, and TIMP-2 mRNA in the monocultured RASFs. RASFs were stimulated with IL-1β (1.0 ng/ml) for 48 hours in the presence or absence of EAAS (100 μg/mL). IL-1β enhanced the expression of MMP-1 and MMP-3 mRNA in RASFs (*p <* 0.05), but not of TIMP-1 and TIMP-2 mRNA. EAAS inhibited the effects of IL-1β on the expression of MMP-1 and MMP-3 mRNA (*p <* 0.05 and *p <* 0.01, Figure 2). IL-1β also enhanced the expression of COX-2 mRNA in RASFs (*p <* 0.01), but not of COX-1 (data not shown). EAAS inhibited IL-1β-induced expression of COX-2 mRNA (*p <* 0.05, Figure 2).

### Effect of EAAS on IL-1β-induced MMPs, TIMPs, and COX protein expressions in RASFs

**Figure 3:**
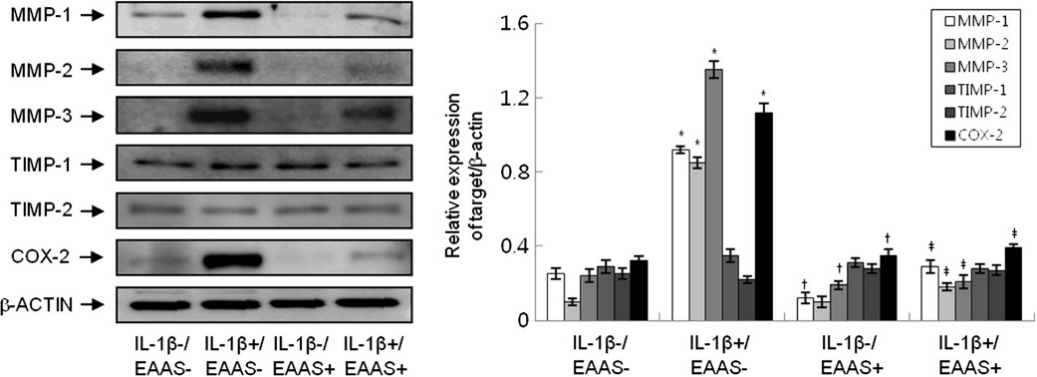
**The protein were extracted from RASFs and analyzed by Western blotting.** IL-1β enhanced the expression of MMP-1, MMP −3, TIMP-1, and COX-2 protein compared with the results without IL-1β. EAAS inhibited the IL-1β-induced expression of MMP-1, MMP-3, and COX-2 protein. Results are presented as mean ± SD. n = 3. *p < 0.05 versus no IL-1β and EAAS, †p < 0.05 versus no IL-1β and EAAS, ‡p < 0.05 versus IL-1β without EAAS.

To further evaluate the expressions of MMPs, TIMPs, and COX proteins in mono-cultured RASFs, we performed Western blotting. RASFs were stimulated with IL-1β (1.0 ng/mL) for 48 hours in the presence or absence of EAAS (100 μg/mL). IL-1β enhanced the expressions of MMP-1 and MMP-3 proteins in RASFs (p < 0.05), but not of TIMP-1 and TIMP-2 proteins, consistent with the results of mRNA expression (Figure 3). EAAS inhibited IL-1β-induced expressions of MMP-1 and MMP-3 proteins (p < 0.05,). IL-1β also enhanced the expression of COX-2 protein in RASFs (p < 0.01), but not of COX-1 (data not shown). EAAS inhibited IL-1β-induced expression of COX-2 protein (p < 0.05). EAAS also significantly decreased expressions of MMP-1, MMP-3, and COX-2 proteins compared to those in the dimethyl sulfoxide (DMSO) control condition without IL-1β and EAAS (p < 0.05).

### EAAS inhibits IL-1β-induced PGE2 production in RASFs

**Figure 4:**
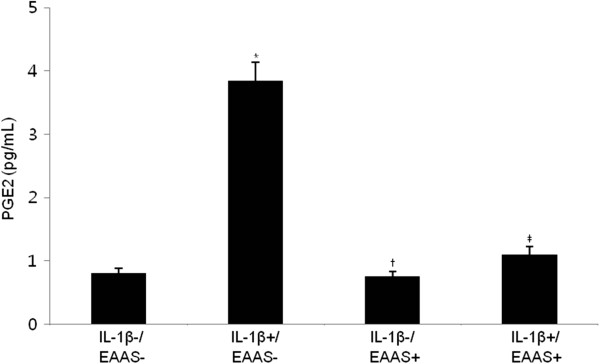
**PGE2 production was inhibited by treatment with EAAS.** IL-1 production of PGE2 by RASFs was evaluated with ELISA kit. IL-1β-induced PGE2 production was significantly inhibited by EAAS. Results are presented as mean ± SD. n = 3. *p < 0.05 versus no IL-1β and EAAS, ‡p < 0.05 versus IL-1β without EAAS.

To confirm the effect of EAAS on the role of IL-1β in PGE2 production by RASFs, we examined the concentration of PGE2 in the culture supernatant. RASFs (1 × 10^4^ cells) were grown in 25 cm^2^ tissue-culture flasks for 48 hours before and after treatment with IL-1β (1.0 ng/mL) and/or EAAS (100 μg/mL). PGE2 production was increased after IL-1β treatment (*p <* 0.05) in comparison to the control, and it was significantly inhibited by treatment with EAAS at 48 hours, as was expected with the results of COX-2 expression (Figure 4).

### Effect of EAAS on IL-1β-induced signal pathways in RASFs

**Figure 5:**
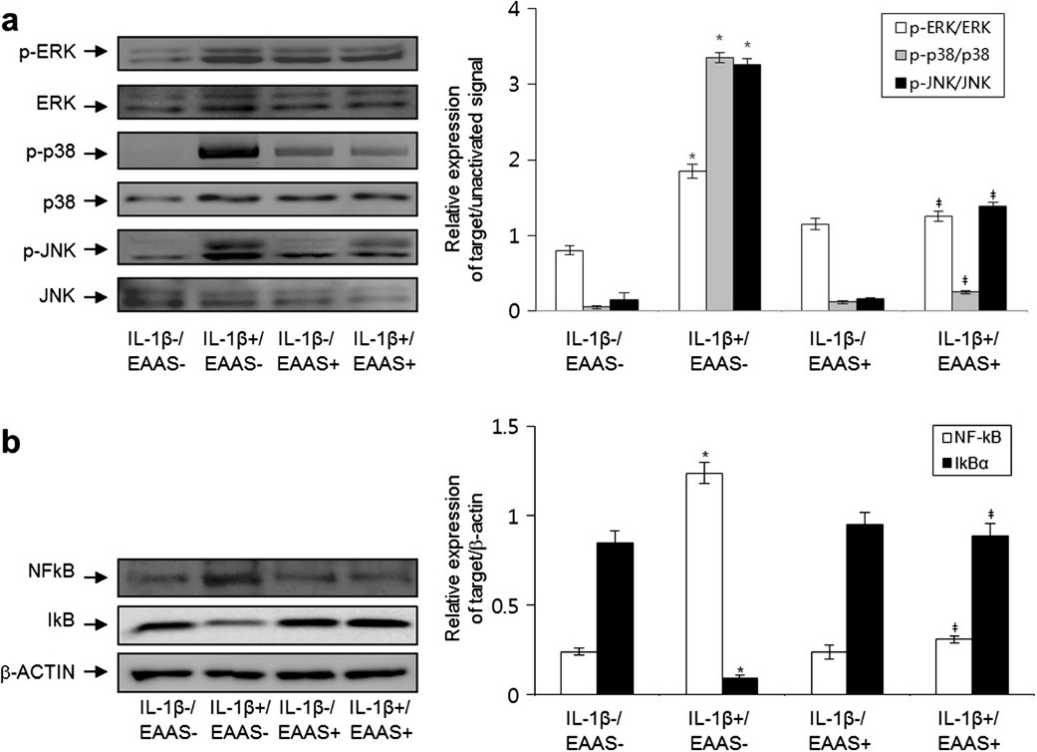
**Effects of EAAS on IL-1β-induced MAPK signal pathways and NF-kB activation in RASFs.** IL-1β enhanced the phosphorylation of ERK, p38, and JNK and EAAS inhibited the IL-1β-induced activation of p38 and JNK **(a)**. IL-1β activated nuclear NF-kB and decreased cytoplasmic IkBα and EAAS inhibited the IL-1β-induced activation of NF-kB **(b)**. Results are presented as mean ± SD. n = 3. *p < 0.01 versus no IL-1β and EAAS, ‡p < 0.05 versus IL-1β without EAAS.

To demonstrate the involvement of the signal transduction and the mechanisms of the effects of EAAS on IL-1β-induced RASFs proliferation, MMPs and COX-2 expression, and PGE2 production, activation of MAPKs and NF-κB were evaluated in the RASFs. IL-1β activated the intracellular MAPKs, including extracellular signal-regulated kinase (ERK), p-38, and c-Jun N-terminal kinases (JNK), and EAAS significantly inhibited the IL-1β-induced intracellular MAPKs activation (Figure 5a). Activation of NF-κB and p65, and degradation of cytoplasmic IkBa was observed in RASFs treated with IL-1β. These effects of IL-1β on NF-κB activation were abrogated by EAAS (Figure 5b). These results indicate that EAAS might inhibit IL-1β-induced proliferation of RASFs, expression of COX-2, and production of PGE2 via intracellular MAPKs and NF-κB pathways.

## Discussion

Since herbal medicines have become popular around the world, pharmacological evidence to help understand the action of these medicines and their underlying mechanisms is needed. Recent therapeutic interest has focused on AS’s cardiovascular, hepatoprotective, antioxidant, and immunomodulatory properties. The active principle of AS has been found to include a number of anti-inflammatory substances [10]. The main chemical constituents of *Angelica* roots are ferulic acid, Z-ligustilide (the main lipophilic component of the essential oil of AS), angelicide, butylidenephthalide, and butylphthalide [11]. Ferulic acid has been demonstrated to exhibit anti-Alzheimer’s disease, antimicrobial, and anticarcinogenic properties. The amounts of ferulic acid and Z-ligustilide in the EAAS were calculated as 3.74 and 15.95 mg/g dry weight of the whole plant, respectively [9]. This component of EAAS also showed higher inhibitory activity of NF-κB transactivation than hexane or water fractions [9]. Furthermore, EAAS inhibits the production of inflammatory mediators alleviates acute inflammatory hazards and protect mice from endotoxic shock [12]. However, the exact components of AS that are involved in anti-inflammatory effects in RASFs are not yet known. Furthermore, there were no reports on the effects of EAAS on the inflammatory reactions, including production of MMPs, COX-2, and PGE2 by RASFs and the mechanisms, which play a crucial role in the pathogenesis of synovitis and articular destruction in RA. We showed here that EAAS inhibits IL-1β-induced cell proliferation, COX-2 expression, and PGE2 production in RASFs by inhibition of activation of the MAP kinases ERK1/2, p-38, and JNK, and NF-κB signaling pathways. These findings suggest that EAAS can be used as a new therapeutic agent for management of RA by decreasing inflammation.

The hyperplasia of synovial fibroblasts is one of the most striking features of RA and is considered to be essential for the evolution of joint destruction in RA [2]. The tumor-like proliferation of RASFs is considered to be the major mechanism for the hyperplasic growth of the RA synovium and eventual destruction of the articular bones and cartilage.^3^ IL-1β plays an important role in the pathogenesis of inflammatory synovitis and joint destruction in RA by inducing the proliferation of fibroblast cell lines [13]. This study showed that EAAS significantly inhibits IL-1β-induced proliferation of RASFs in a dose- and time-dependent manner. This probably means that EAAS would be an ideal treatment for RA. However, further study is required to know the effects of EAAS on RASF proliferation in the absence or presence of stimulating factors and to define the exact mechanism.

It has been well known that both COX-1 and COX-2 are expressed by human RASFs and that the expression of COX-2 is enhanced by proinflammatory cytokines, such as IL-1β [14]. COX-2 converts free arachidonic acid into prostaglandins, including a variety of bioactive products (PGI2, thromboxane A2 (TXA2), PGE2, and PGD2). PGE2, a pleiotropic mediator of inflammation, provides certain homeostatic functions, but its excessive production in the joints of RA patients is associated with many pathologic processes, and plays a critical role in eliciting the signs and symptoms of inflammation [15]. This study found that EAAS inhibits both IL-1β-induced COX-2 protein expression and PGE2 synthesis dose-dependently over time. Thus, further studies are also needed to identify the EAAS components that are responsible for the results of our study. It is also necessary to demonstrate the above effects on inflammatory joint disease with *in vivo* systems, such as animal models of RA and collagen-induced arthritis (CIA).

NF-κB and MAPKs participate in inflammation and destruction of joints in RA. It is known that inactive NF-κB normally binds to IkB in the cytosol, and NF-κB can be activated by proinflammatory cytokines, IL-1β and TNF-α [16]. JNK, p38, and ERK are expressed in cultured RASFs and are readily activated by IL-1β [17]. Prostaglandins have also been described as being under the influence of p38 MAPK [18]. This has been confirmed in a study in which it was reported that glucocorticoids destabilize COX-2 mRNA by inhibiting the p38 MAPK route [19]. Numerous studies have demonstrated that inhibitors of MAPKs or NF-κB decrease synovial inflammation, bone destruction, and cartilage damage in animal models of arthritis, including adjuvant arthritis in rats and CIA in mice [20]. To identify the mechanisms of the effects of EAAS on IL-1β-induced proliferation of RASFs, expression of COX-2, and production of PGE2, the activation of the MAPKs and NF-κB was examined. This study showed that EAAS inhibits the IL-1β-induced activation of NF-κB and phosphorylation of ERK, p38, and JNK. However, further studies are needed to identify the very important EAAS components and the mechanism by which EAAS inhibits the effects of IL-1β on RASFs. Further investigation is also needed to discern how EAAS suppresses NF-κB activation, and to identify the components of NF-κB that are suppressed and the kind of intracellular signaling factors specifically or directly involved in the effect of EAAS on the proliferation and PGE2 production in RASFs.

## Conclusions

This is the first study to report that EAAS can inhibit the IL-1β-induced proliferation of RASFs, expression of COX-2, and production of PGE2. This study also showed that EAAS inhibits the activation of NF-κB and phosphorylation of MAPKs pathways. Taken together, these findings suggest that EAAS may be useful in the treatment of inflammatory diseases, including RA. However, further studies are required to identify the exact mechanism underlying the inhibition of synovial cell proliferation and inflammatory reactions, and to find the active components in the EAAS.

## Methods

### Preparation of crude extract of *Angelica sinensis*

AS was collected from Jiri Mountain in South Korea and plant material was identified by an authority at the Rheumatology Laboratory and Research Center for Pulmonary Diseases, Chonbuk National University Medical School, Jeonju, Jeonbuk, South Korea. In total, 10 g of AS was extracted with 300 ml of 95% ethanol at 50°C for 3 hours twice. The total crude extract was evaporated under vacuum to yield a residue, and then the residue was suspended in 90% ethanol and successively partitioned with hexane, benzene, trichloromethane, ethyl acetate, n-butanol and water fractions, consecutively as described [21]. The ethyl acetate fraction was used in this study after well drying the fraction. And we used kaempferol 0.852 ug and decursin 2.116 ug in 1 mg/mL of extract by quantifying the content.

### Reagents and antibodies

Recombinant human IL-1β was purchased from R&D system (Minneapolis, Minnesota, USA). Monoclonal antibody (mAb) against MMP-1, MMP-3, tissue inhibitor of metalloproteinase (TIMP), and COX-2 was purchased from Santa Cruz Biotechnology (Santa Cruz, CA, USA). mAb against extracellular signal-regulated kinase (ERK), phosphorylated ERK (p-ERK), C-jun N-terminal kinase (JNK), phosphorylated JNK (p-JNK), p38, p-p38, nuclear factor κB (NF-κB) (p65), nuclear factor of kappa light polypeptide gene enhancer in B-cells inhibitor, alpha (IκBα) and β-actin were purchased from Cell Signaling (Beverly, MD, USA). Fetal bovine serum (FBS) was obtained from Gibco BRL (Life Technologies, Grand Island, NY, USA).

### Isolation and culture of RASFs

Synovial tissues obtained at the time of total knee arthroplasty in patients who fulfilled the American College of Rheumatology Criteria for RA [22], as previously described [23]. Synovial fibroblasts from passages 3–7 were used for each experiment and were morphologically homogeneous and had the appearance of RASFs with typical fibroblastoid configuration under inverse microscopy. The purity of the cells was tested by flow cytometry using phycoerythrin (PE)-conjugated anti-Thy-1 (CD90) or anti-CD14 and fluorescein isothiocyanate (FITC)-conjugated anti-CD3 mAb (BD Pharmingen, San Diego, CA). Informed consent was obtained from all patients, and the study protocol was approved by the Chonbuk National University Hospital Ethical Committee.

### RNA Isolation and semiquantitative RT-PCR of COX, MMPs and TIMP

Total RNA was extracted from cultured cells using the TRIsol reagent (Invitrogen, Carlsbad, CA, USA) following the manufacturer’s instructions. One microgram of RNA was reverse-transcribed using Maxime RT Premix Kit (iNtRON Biotechnology, Korea). cDNA was amplified using the following primer sets: COX-1 (sense) 5′-GCT ATT CCG GCC CCA ACT-3′ (antisense) 5′-GAT GAA GGT GGC ATT GAC AAA CT-3′, COX-2 (sense) 5′-TCC TTG CTG TTC CCA CCC ATG-3′ (antisense) 5′-CAT CAT CAG ACC AGG CAC CAG-3′, MMP-1 (sense) 5′-GAA GGA GAT GAA GCA GCC CAG ATG T-3′ (antisense) 5′-CAG TTG TGG CCA GAA AAC AGA AGT GAA A-3′, MMP-3 (sense) 5′GAC ACC AGC ATG AAC CTT GTT-3′ (antisense) 5′-GGA ACC GAG TCA GGA CTA TG-3′, TIMP-1 (sense) 5′-CCT TCT GCA ATT CCG ACC TCG TC-3′ (antisense) 5′-CGG GCA GGA TTC AGG CTA TCT GG-3′, glyceraldehyde 3-phosphate dehydrogenase (GAPDH) (sense) 5′-ACC ACA GTC CAT GCC ATC AC-3′ (antisense) 5′-TCC ACC ACC CTG TTG CTG TA-3′. PCR products were electrophoresed by using 1% agarose gels and visualized by staining with ethidium bromide. Densitometric analysis was performed on the relative intensity of each band using the Multi Gauge program, version 3.0 (Fuji film, Tokyo, Japan).

### Cell viability analysis

Cell viability was determined by a cell counting kit-8 (CCK-8; Dojindo Laboratories, Japan) according to the manufacturer’s instructions. Briefly, 2-(2-methoxy-4-nitrophenyl)-3-(4-nitropenyl)-5-(2,4-disulfophenyl)-2H-tetrazolium (CCK-8) was reduced by dehydrogenases in cells to yield an orange-colored product (formazan) [24]. The amount of the formazan dye generated by dehydrogenases in cells was directly proportional to the number of living cells. RASFs (1 × 10^5^ cells per well in complete RPMI-1640 media in a 96-well plate) were cultured in 200 mL medium per well without antigen stimulation in the presence or absence of 100 μg/mL EAAS for 2 days. CCK-8 (20 mL) was added to each well of the plate and the cells were incubated for 2–3 hours. The absorbance was measured at 450 nm using a microplate reader.

### Assay of PGE2 production

RASFs were grown in 25 cm^2^ tissue-culture flasks for 48 hours before treatment. After washing with PBS (pH 7.4), cells were pretreated with IL-1β (1.0 ng/mL) or EAAS (100 μg.mL) at 37°C for 48 hours in DMEM containing 10% (v/v) FCS in an atmosphere of 5% CO_2_. The culture supernatant described above was collected at 2 days. The level of PGE2 in the medium was determined by ELISA kit (R&D Systems) in accordance with the instructions of the manufacturer.

### Immunoblotting

RASFs (1 × 10^6^ cells) were seeded on 100-mm culture dishes and harvested in phosphate buffered saline (PBS) after stimulation as described above. Cells were lysed in lysis buffer containing 50 mM Tris-CL, 150 mM NaCl, 5 mM EDTA 1% Triton X-100, 1 mM sodium fluoride, 1 mM sodium vanadate, 1% deoxycholate, and protease inhibitors. To determine the membrane COX-2 expression on RASFs, cell membranes were prepared from isolated RASFs, as described previously [25]. To analyze NF-kB (p65), nuclear extract was prepared using a previously described method [23]. To determine the cytoplasmic IkBa, cytoplasmic extracts were prepared from isolated RASFs as described previously [23]. The protein concentration was determined by the Bio-Rad protein assay regent (Bio-Rad Laboratories, USA). Samples (50 mg) were prepared with the four volume of 0.5 M Tris buffer (pH 6.8) containing 4% SDS, 20% glycerol and 0.05% bromophenol blue at 95°C for 5 min. SDS-PAGE was performed in 10% slab gel. Proteins were transferred to nitrocellulose paper. The membrane was washed in blocking buffer (10 mM Tris–HCl pH 8.0, 150 mM NaCl, 5% fat-free milk) for 60 min at room temperature with shaking and then washed with TBST (TBS, 0.01% Tween 20). Primary antibodies (10 mg/ml) against MMP-1, −3, TIMP-1, −2, COX-1, −2, ERK, p-ERK-1/2, p-38, p-p38 MAPK, JNK, p-JNK, NF-kB (p65), IkBa and β-actin was incubated at 4°C for 4 hr. The secondary HRP-conjugated antibody was goat anti-mouse IgG (Stressgen Bioreagents, Ann Arbor, MI, USA). Reactive proteins were detected using enhanced chemiluminescence (ECL, Amersham Life Sciences, Arlington, IL, USA) using Fuji film LAS-3000 (Tokyo, Japan).

### Statistical analysis

All data were expressed as the mean ± SD of triplicates and all data were analyzed by the SPSS 18.0 program. Group mean values were compared by Student’s t test or ANOVA as appropriate. The significance of difference was defined as *p* values < 0.05, or *p* values < 0.01.
